# The hatred of all against all? Evidence from online community platforms in South Korea

**DOI:** 10.1371/journal.pone.0300530

**Published:** 2024-05-06

**Authors:** Jeong-Woo Koo, Chan S. Suh, Jin Won Chung, Kyung-Ah Sohn, Kyungsik Han

**Affiliations:** 1 Department of Sociology and the Social Innovation Convergence Program, Sungkyunkwan University, Seoul, Republic of Korea; 2 Department of Sociology, Chung-Ang University, Seoul, Republic of Korea; 3 Department of Sociology, The Catholic University of Korea, Seoul, Republic of Korea; 4 Department of Software and Computer Engineering, Ajou University, Suwon, Republic of Korea; 5 Department of Data Science, Hanyang University, Seoul, Republic of Korea; CCET: Chandigarh College of Engineering and Technology, INDIA

## Abstract

**Background:**

Over several years of recent efforts to make sense and detect online hate speech, we still know relatively little about how hateful expressions enter online platforms and whether there are patterns and features characterizing the corpus of hateful speech.

**Objective:**

In this research, we introduce a new conceptual framework suitable for better capturing the overall scope and dynamics of the current forms of online hateful speech.

**Methods:**

We adopt several Python-based crawlers to collect a comprehensive data set covering a variety of subjects from a multiplicity of online communities in South Korea. We apply the notions of marginalization and polarization in identifying patterns and dynamics of online hateful speech.

**Results:**

Our analyses suggest that polarization driven by political orientation and age difference predominates in the hateful speech in most communities, while marginalization of social minority groups is also salient in other communities. Furthermore, we identify a temporal shift in the trends of online hate from gender to age based, reflecting the changing sociopolitical conditions within the polarization dynamics in South Korea.

**Conclusion:**

By expanding our understanding of how hatred shifts and evolves in online communities, our study provides theoretical and practical implications for both researchers and policy-makers.

## Introduction

Recently, online hateful speech has been receiving considerable attention among diverse stakeholders in academia, government, and information and communications technology (ICT). With greater tolerance of the freedom of speech in the digital era, hateful and contempt expressions are often perceived as falling within the right’s protective ambit. Nonetheless, various actors have called attention to the need to reveal the harms of online hateful speech and consider regulating online speech with proper moral and legal tools, although democracies fiercely disagree on this critical issue [1].

What are the variety and the extent of hateful speech? How does hateful speech evolve? These are important questions worth considering in examining how to carve out proper intervention measures against such new challenges facing the digital society. Over several years of recent efforts to make sense and detect online hate speech, we still know relatively little about (a) how hateful expressions enter online platforms and (b) whether there are patterns and features characterizing the corpus of hateful speech.

To answer these questions, we introduce a new conceptual framework suitable for better capturing the scope and dynamics of the current forms of online hateful speech. While the widespread view of hatred attaches to social hierarchies associated with status distinction between majority and minority groups, our new account links hateful speech not only to these hierarchies but also social polarization between two competing groups. Polarization refers to dichotomies that require taking a side, such as left-wing versus right-wing political proponents, men versus women arguing for their superiority, or older versus younger citizens where each proclaims its superiority over the other. Marginalization, meanwhile, refers to being disregarded or openly harmed because of belonging to groups such as religious, racial or ethnic, or sexual minorities or because of being disabled. We work with the notions of *marginalization* and *polarization* to detect and understand the patterns and shifts of online hateful speech by analyzing a hate corpus we newly compiled from South Korean online social media during the period 2015–2022.

Social media is depicted as facilitating the person-to-person diffusion of expressions, and therefore a vast majority of research focused on the roles of social media platforms, such as X (formerly Twitter) or Facebook, in spreading hateful and contempt expressions [2–6]. In contrast, fewer attention has been paid to the specialist and less conventional—sometimes ideologically driven—online platforms, such as Reddit, Gap, 4Chan, or Stormfront. This is a serious oversight given that specialist online communities serve as a reservoir within which they create, modify, and spread hateful expressions to the society. Individuals engaging in these online communities share a common sense of belonging, and amorphous groups of individuals tend to share hateful expressions as a way to strengthen their common identity. Furthermore, these communities often serve as echo chambers in which biased opinions and hateful expressions are reinforced among participants. Considering the few recent examinations of these less conventional platforms see their roles as important channels for online users to generate and spread hatred [7–9], we bring attention to both generalist and specialist types of online communities in our data corpus.

The lack of sufficient attention to specialist online communities is primarily attributable to technical difficulties in gathering comprehensive data from their layouts subject to permanent change. To fill in this gap, we implemented several Python-based crawlers corresponding to a multiplicity of South Korean online communities and used them to collect an unprecedented data set of communications in online communities throughout the country during the period from 2015 to 2022. In general, we adopted an inclusive data collection strategy by covering a variety of subjects—politics, gender, age, race/ethnicity, religion, sexuality, and disability—and employed a keyword-based approach to collect over 396,496 postings and comments that could be considered hateful, contemptuous expressions in the South Korean context from 11 of the most frequently visited online platforms in South Korea.

South Korea provides an excellent laboratory for investigating online hateful speech because of the myriad of technological, political, and social conditions associated with the country: (a) the fastest Internet connectivity in the world coupled with the debut of large platform companies and their influences, (b) the legacy of contentious politics encouraging status competition and the subsequent political polarization, and (c) acute and rampant social conflicts making their way to online spaces. Yet scant attention has been paid to the breadth and depth of what has shaped the nature of online social platforms.

Introducing a new conceptual framework to identify the patterns and changes in hateful expressions over time and across communities, our analysis of the newly compiled South Korean hate speech corpus shows that both marginalization and polarization effectively capture the web of networks consisting of members who form and maintain their identities using hateful languages. More specifically, our analyses suggest that polarization driven by political orientation and age or generational differences predominates in hateful communities though we also note the diffusion of hatred against minority groups, including sexual minorities and people with disabilities. We find much evidence that a wide spectrum of hateful expressions arose and expanded in varying degrees. Of particular importance was a temporal shift in the trends of hatred from gender to age based, reflecting the sociopolitical conditions during the observational period.

## Theoretical background

Social media provides access to information and participation, revitalizing the public sphere and supporting the formation of civic spaces. With the pervasiveness of ICT and the ways it facilitates information acquisition and public discussion, social media platforms offer favorable conditions for publishing citizens’ own opinions and challenging others’ viewpoints, thereby broadening the basis of democratic engagement [10–13]. Some online platforms facilitated mass mobilizations and public protests, showcased by the Arab Spring and the Occupy movement, that even led to some democratic reforms [14–18].

Whereas many heralded the rise of online platforms as having the potential to contribute to democracy and human well-being, others began to challenge this normative conceptualization of social media by providing ample evidence of its opposite effects. Hate groups grow in prominence, and their expressions of hatred and contempt assault the human dignity of vulnerable populations. Like-minded users band together to create echo chambers in which they and their peers reinforce each other’s opinions, attitudes, and expressions [19–25]. Group polarization theory argues that echo chamber effects move large groups toward more extreme positions and often lead them to nurture biases and stereotypes and spread misinformation and hateful expressions [2, 26]. As the digital transition has intensified and freedom of expression has so often been misused, the need to address online hate speech is only growing.

Why does hateful speech exist and why does it matter? The current account views hateful speech as both a reflection and the source of social hierarchies [27]. This account posits that hateful and contemptuous speech targets vulnerable groups, undermines their human dignity, and stigmatizes them as out-group members, perpetuating social hierarchies. Both legal and social science literature on hatred rely on this widespread view, which guides questions regarding how deeply the myriad forms of defamation have penetrated online platforms. It is not a coincidence that race/ethnicity, religion, nationality, and sexual orientation have been the primary targets of hatred in the literature on hatred in general and online hateful speech in particular.

Our account expands this influential view into a new conceptual framework in which we treat hatred as increasingly relevant to social polarization. We explore the possibility that hate speech increasingly reflects and maintains polarization between competing social groups.

Hatred should not be narrowly understood to be limited to a relationship between majorities and minorities but needs to incorporate even the construct of majority against majority. Political ideology, age groups, and gender are increasing sources of online hate speech, alerting even many societies that emphasize respect for rights to be wary of new forms of hatred.

Our account relies on a sociological perspective that accentuates a social and cultural construction of the hateful rhetoric. As Kennedy et al. [28] observed, “hate speech is fundamentally embedded within the existing cultural and social context in which it occurs”. Consequently, the weights assigned to each category of hatred and their temporal shifts that we focus on here might be largely context specific, reflecting political and cultural dynamics that are central to South Korea. Considering, for example, the cultural legacy of South Korea as a monolithic country rooted in the same blood and ancestry, there is less likely to be hatred based on racial and ethnic attributes than there might be in the multicultural, multiethnic settings countries such as in the West or in Africa.

In Korea, the social environment in which men have higher rates of labor participation and higher pay than women indicate gender as a likely subject for hatred, especially after women began making rapid achievements in social status. These cultural and institutional contexts highlight the need for a more general sociological investigation addressing the patterns and dynamics of cyberhate. Another pathway toward a culturally sensitive sociological understanding of hate speech might be to shed light on the local language and examine the words or the rhetoric that could be perceived as insulting or dehumanizing. Toward that aim, we compiled a list of words or phrases likely to be associated with hateful expressions on online social media platforms.

## South Korean hatred in action

Our culturally and institutionally sensitive approach permits us to canvass several categories of hatred and identify keywords corresponding to each category. If the hateful rhetoric is embedded in social and cultural contexts, we should expect different manifestations of defamation from country to country and society to society. In the United States for instance, Mollas et al. [29] identified gender, race, national origin, disability, sexual orientation, and religion as domains of hatred, although they did not report on the prevalence or relative weights of these categories. Considering the South Korean context, we combine race and national origin and add political hatred to broaden the scope of hatred and more effectively capture the extent to which hate speech prevails in South Korea.

In providing a comprehensive overview of how hateful expression has changed over time in South Korea, our conceptual framework allows us to identify polarization that brings to the fore the three forms of social conflicts, i.e., political affiliation, gender, and age. Identifying marginalization as the other pillar of our framework, our research detects how much hatred is directed toward minority groups who already experience myriad forms of defamation and discrimination. Considering the comprehensive nature of our data, we indirectly measure the symptoms of polarization and marginalization, respectively, by examining the target of hatred expressions. More specifically, the dynamics of polarization is accompanied by two groups generating and sharing hatred keywords against each other. Likewise, minority groups are likely to become the target of hatred expressions by the majority group in the process of marginalization.

Based on the pillars of polarization and marginalization, the first area of hate speech we address is political hatred. Hate recently became the new currency of politics across the world with hateful aggression being preferred over courteous treatment among political opponents. Hate finds its expressions in countries that experienced heightened political polarization. Increased polarization leads to antagonism towards political opponents, turning nonpolitical issues such as climate change into politicized agendas and generating misinformation and fake news [30, 31]. This phenomenon holds in South Korea as well given its de facto two-party system coupled with a strong presidency; political opponents create hate stories with derogatory expressions and use them as weapons for defaming each side of the ideological spectrum. For the data collection, we choose three widespread and representative keywords relevant to this important dimension of hatred in South Korea: (a) *jwappal*, a pro-North Korean communist, (b) *sukkol*, an obstinate far-right member, and (c) *daekkaemun*, one expressing steadfast and uncritical support for former President Moon Jae-in. Given the escalated polarization between progressives and conservatives, users even justify the use of these hateful keywords by noting that it is rather hypocritical not to use those terms to each other.

Hatred spurred by age differences is also a byproduct of social polarization, and age-based hateful speech appears to be a major form of online hateful rhetoric in South Korea. Rooted in Confucian ideology privileging seniors and the recent problem of low birth rates burdening younger generations with future social security, the country is increasingly polarized. An unprecedented unemployment rate for young people is depriving them of their rights to work and adequate standard of living, exacerbating their antagonism toward the establishment, authority, and the older generation.

Additionally, the rapid digital transition has made older and elderly adults vulnerable to stigmatization for not adopting quickly, and in response, derogatory and defamatory expressions targeting the younger generation are commonplace online [32]. Consequently, we aimed to assess the degrees of hatred targeting older versus younger people in online communities with searches for the following three keywords: (a) *teulttak*, insulting old people by calling them dentures, (b) *jaemmin*, a clueless, ignorant child, and (c) *yuchung*, larva, a term used for humiliating a child.

Gender-based hatred is the other major form of online hate speech around the world including South Korea. Gender-based hatred tends to be primarily men’s misogynistic aggression toward women. With the influence of feminism, and the ongoing tug of war between young men and women online, however, misandry has received much attention as well. In response to misogynistic expressions on the Internet, female groups have mirrored the language by spreading anti-male hate expressions [33]. In the aftermath of the Me-Too movement circa 2018 and the backlash movement, gender became a contentious topic between men and women. The keywords that represented this bitter online competition between men and women, included (a) *kkolpemi*, an insult to radical feminists, (b) *hannamchung*, comparing Korean men to bugs bug, (c) *megallyeon*, the other derogatory expression for women and feminists, and (d) *gaejeossi*, an insult to middle-age men. Due to the contentious nature around gender politics, the usage of these hateful terms is justified as a political tactic to attack the other group.

Turning to hatred toward minority groups through the lens of marginalization, we analyzed the four social groups vulnerable to aggression, contempt, and discrimination: racial and ethnic minorities, sexual minorities, religious minorities, and the disabled. Whereas the discourse and treatment of political groups, age groups, and gender tend to reflect social polarization, we maintain that the domains of race/ethnicity, sexuality, religion, and disability reflect remain the vulnerable subjects of marginalization that subjects them to aggression.

Regarding race and ethnicity, South Korea is ethnically homogeneous, with an exceptionally low score on the ethnic fractionalization index [34]. Online hate towards racial and ethnic minorities has become prevalent, however, as the number of marriage-migrant women and migrant workers has gradually increased [35]. The debate over accepting Yemeni refugees in 2008 also spiked racial hatred, especially towards male Muslims. Here we searched for three common insults: (a) *jjanggae*, toward Chinese migrants, (b) *ttongnama* toward Southeast Asian migrants, and (c) *joseonjoksaekki* toward Chinese Koreans.

As for sexuality, the long legacy of Confucianism in South Korea has led to antipathy toward individuals who do not follow traditional familial values, such as sexual minorities. Over the past 25 years, however, sexual minorities have not only mobilized through activism but also become much more visible to the public through movement tactics such as queer parades [36]. In our data set, we searched for terms of hate toward sexual minorities: (a) *geinom* referring to gay men, (b) *ttongkkochung*, humiliating gay men, and (c) *rejeunyeon*, insulting lesbians.

We also addressed online hate toward religious minorities. Although Protestantism, Buddhism, and Catholicism are the three major religions in South Korea, approximately half of the population self-report as non-religious [37]. Therefore, religious groups often become the target of hatred from the public. During the COVID-19 pandemic, for example, Protestants came under attack because some churches ignored social distancing policies and continued holding religious gatherings. Insulting expressions toward Muslims have also been increasing as the Muslim population continues to grow. The keywords used in our analysis include (a) *gaedok*, humiliating Christian believers, (b) *gaeseullam*, insulting Muslims, and (c) *jotseullam*, another insult to Muslims.

Finally, we included disability as a target of online abuse because people with disabilities have long been targets of verbal abuse. Stereotypes and hatred toward people with mental and physical disabilities have deep roots in countries around the world including South Korea and have held even through industrialization and democratization because of ongoing societal distinctions between normal and abnormal [38]. The two keywords we searched for were (a) *jeongsinbyeongja*, a derogatory word for people with mental illness and (b) *jangaeinsaekki*, another humiliating term for the disabled.

## Data and methodology

### Online communities

To sample documents for online hateful speech, we selected social media platforms or online communities that are widespread in South Korea. We took advantage of Alexa Internet, which provides global rankings for over 30 million websites according to the number of daily visitors and page views [39]. For South Korea, Alexa Internet ranked the 500 most popular websites in order. From this list, we selected eight voluntary specialist communities that were neither government websites nor corporate or commercial shopping websites: DC Inside (22), FM Korea (30), Ruliweb (38), Inven (56), Clien (62), Ppomppu (101), Ilbe Storage (192), and Humoruniv (433) The actual rank of each community is indicated in parentheses. We removed two online communities, Instiz and Todayhumor, from the list during the data collection stage because there were comparatively few hateful expressions in these communities, making them not particularly representative. In addition, we added three popular generalist online platforms, Naver Café and Naver Band and Daum Café, that allow users to create online communities. These platforms are operated by Naver Corporation and Kakao Corporation, respectively, the two most dominant platform companies in South Korea. With this comprehensive list of generalist and specialist communities, we are able to examine which types of online communities serve as a reservoir within which hateful expressions are created, modified, and spread to the society.

Because social platforms typically comprise many individual communities on their platforms, it is hard to generalize the average characteristics of primary users for each community. Indeed, researchers have found that online communities display variation in their overall characteristics, from political conservatives (e.g., Ilbe Storage and FM Korea) to liberals (e.g., Clien and Ppomppu) and from younger (e.g., FM Korea) to older populations (e.g., Naver Band) [40–42]. Our list of communities appears to be comprehensive in that we covered the most popular online communities in South Korea; systematically sampling a wide range of platforms allowed us to generate a more representative data set.

### Data collection

For the sampling strategy, we searched keywords in 11 online communities. Searching for keywords is not only a conventional way of collecting massive data on hate speech but also an efficient strategy for identifying and revealing which hateful subjects are prevalent compared to others [43]. While keyword searches have its limitations such as failing to capture the nuanced contexts within which hateful expressions are made, keyword-based analysis allows us to explore over a long timespan across multiple communities.

An ontological dictionary is needed to extract keywords from a given text, and therefore, we initially collected all keywords that appeared in previous studies on online hate in South Korea [32, 44–49]. We monitored online communities for about three months to determine if any terms were missing from our initial list of keywords. Second, as indicated in the previous section, we selected several hateful keywords for each category of online hate most frequently used in online communities. Finally, we collected data from 11 online communities using web crawlers. This was a complicated process because we aimed for comprehensive multiyear data sets from multiple community websites with different layout structures, subtopic boards, and subcommunities Regarding old online communities, such as Ruliweb, our data covered a 20-year span from 2001 to 2021. If the layout structure of a given website changed dramatically, however, we were unable to collect data since its founding. The shortest time span that we cover was for DC Inside, from 2015 to 2021 only. Although focusing on keywords meant that we might have failed to capture hateful expressions that did not directly use any of our keywords, it was still an efficient approach for collecting a comprehensive data set on online hate—especially if the list of keywords successfully captured the nuances within various hateful expressions.

After collecting raw data from online communities, we cleaned the data through three phases of data preprocessing: (a) deduplication, (b) homonym removal, and (c) stopword removal. In the first phase, we deduplicated the content of the posting when the title and the content of a posting were identical; we maintained others’ comments in these duplicated postings. In the second phase, we removed cases where the keywords identified as hateful expressions were not used in a hateful context. This was quite common when the given keywords were used in traditional contexts or as hateful slang. To distinguish between the two, we employed Kang et al. [32]’s deep learning-based hate detection model. In doing so, we set a threshold of 0.9 to minimize unnecessary data loss. In the final phase, we extracted keywords from the original text using Soynlp—a library of unsupervised natural language processing techniques available in Python. Soynlp is appropriate for extracting newly coined words from Korean texts where the meaning structures of sentences may not be divided by spaces [50]. After normalizing words using Soynlp, we could generate a ranking of word frequency among hateful keywords. After implementing data preprocessing, 396,496 online posts and comments remained. Using the cleaned data, we constructed a list of the most frequently used hateful keywords linked to each of the seven categories of online hate It is possible that active bots may skew the finding from our study. While active bots are indeed prevalent in global online platforms such as X and Facebook, we are confident that South Korean online communities are relatively free from these bots. Since online communities in South Korea are structured in a bulletin board format, online community users are able to detect the automated and replicated posts. Our research team has also manually coded the texts that we collected from these platforms, and we have not found any suspicious posts that are generated automatically.

## Results

**Fig 1:**
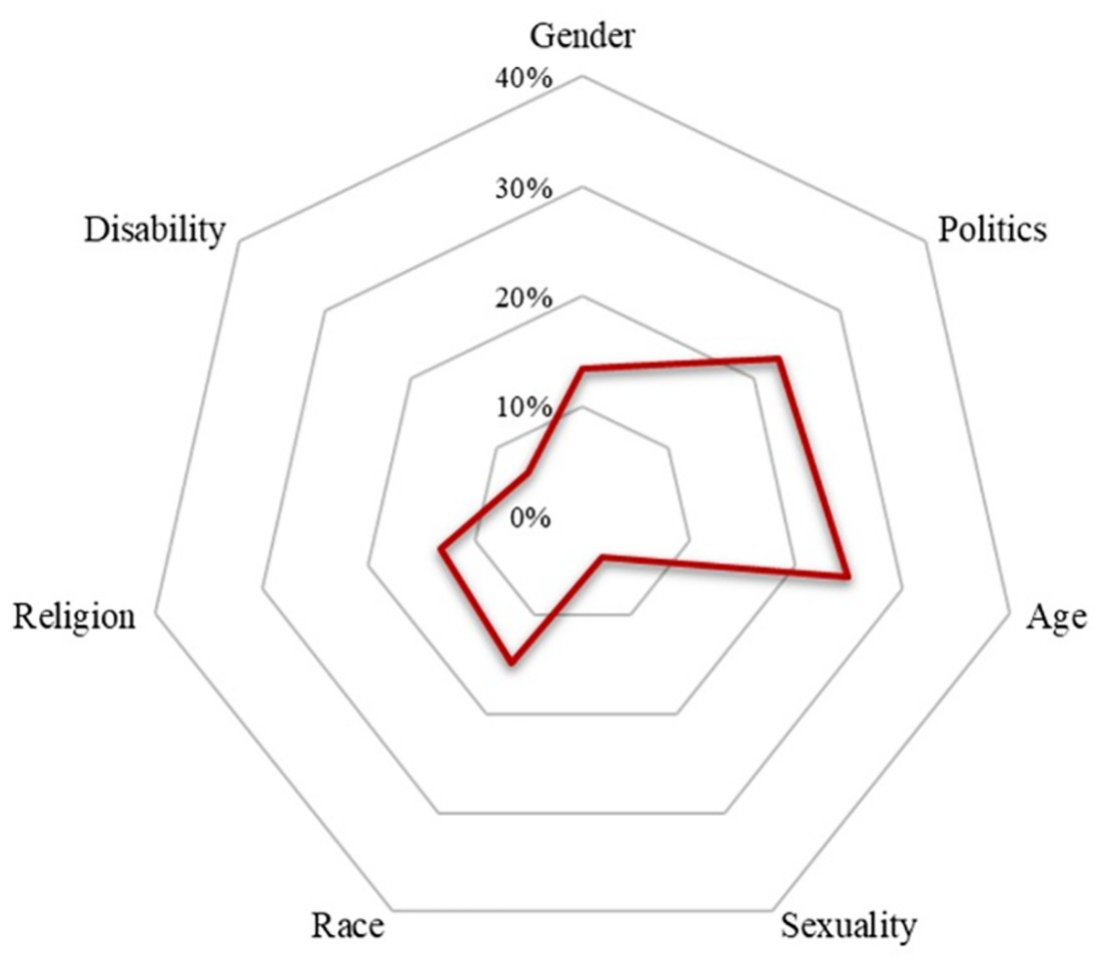
Hateful expressions by category.

We examined the landscape and prevalence of online hatred in South Korea using multiyear comprehensive data compiled from 11—both generalist and specialist—online communities. To begin, we visualized the proportions of hateful expressions in the seven domains of hatred using the radar chart we present in Fig 1; radar charts are suitable for displaying the distribution of multiple variables in a single map. The chart in Fig 1 reflects the total number of posts that included hateful keywords for a given category divided by the total number of all hateful posts in our data during the period from 2015 to 2022.

As shown in Fig 1, online hatred related to age (25.0%) and politics (22.9%) was more prevalent than hatred in any of the other categories, followed by race/ethnicity (14.9%), gender (13.4%), and religion (13.3%). In contrast, the domains of disability (6.4%) and sexuality (4.2%) were substantially less prevalent than others. These findings suggest that both social polarization and marginalization are salient in the the ways in which hatred entered into online social platforms. The findings further suggest that hateful expressions involving social polarization–reflected in political views, age differences, and gender division–is more prevalent than marginalization connected with the suffering of vulnerable social groups–such as racial, religious, and sexual minorities and the disabled. Taking these findings together, the radar chart in Fig 1 reflects the variety and extent of hateful expressions in South Korea. Table 1 displays the frequency and proportion of hateful postings and comments according to the selected keywords.

**Table 1:** Description of hateful keywords by category.

Hateful Expression			Frequency	Percent
Category	Keywords	Meaning		
Gender	꼴페미 (*kkolpemi*)	This is a compound word of “*kkol*” from a narrow-minded person, “*kkoltong*” and “*pemi*” from “feminists.”	24,572 (6.1%)	13.3%
	한남충 (*hannamchung*)	This word means “Korean male bug.” It is a compound word of “*hannam*” from a derogatory term for Korean males and “*chung*” from a bug.	14,537 (3.6%)	
	메갈년 (*megallyeon*)	This is a compound word of “*megal*,” meaning users of Megalia—an online community for radical feminists who use anti-male expressions as a mirroring tactic—and “*nyeon*,” a degrading word for females.	8,144 (2.0%)	
	개저씨 (*gaejeossi*)	This word refers to a rude adult male. It combines “*ajeossi*,” meaning an adult male, with “*gae*,” meaning a dog.	6,527 (1.6%)	
Politics	좌빨 (*jwappal*)	This is a derogatory term referring to a pro-North Korean communist.	42,519 (10.5	23.3%
	수꼴 (*sukkol*)	This is a derogatory term referring to a member of the radical right. It is a compound word of “*sugu*,” meaning a conservative, and “*kkoltong*,” meaning a narrow-minded person.	27,282 (6.8%)	
	대깨문 (*daekkaemun*)	This a derogatory term for someone who unconditionally and uncritically expresses support for President Moon Jae-in, who was in power from 2017 to 2022.	24,198 (6.0%)	
Age	틀딱 (*teulttak*)	This is a derogatory and insulting word describing older people. The word “*teul*” means dentures, artificially made teeth, and “*ttak*” describes the sound of dentures clicking.	56,252 (14.0%)	14.9%
	잼민 (*jaemmin*)	This term is used to describe a clueless, ignorant child. An antonym for “*jaemmin*” is “*teulttak*,” a derogatory word for old people.	26,052 (6.5%)	
	유충 (*yuchung*)	This word originally meant “larva.” It is used as a derogatory expression for a child.	17,129 (4.2%)	
Race/Ethnicity	짱깨 (*jjanggae*)	This is a degrading expression to describe China and its people.	48,396 (12.0%)	24.7%
	똥남아 (*ttongnama*)	This is a degrading expression to describe Southeast Asia and its people.	9,755 (2.4%)	
	조선족새끼 (*joseonjoksaekki*)	This is a compound word of “*joseonjok*,” meaning a Korean-Chinese, and “*saekki*,” a degrading term for a male.	2,032 (0.5%)	
Sexuality	게이놈 (*geinom*)	This is a derogatory expression for gay men. The term “*gei*” refers to a gay man, and “*nom*” is a derogatory term for males.	3,822 (0.9%)	4.2%
	똥꼬충 (*ttongkkochung*)	This is an insulting and contemptuous term for gay men. The term “*ttongkko*” means asshole, and “*chung*” means a bug.	9,610 (2.4%)	
	레즈년 (*rejeunyeon*)	This is a derogatory expression for lesbians. The term “*rejeu*” refers to lesbians, and “*nyeon*” is a derogatory term for females.	3,326 (0.8%)	
Religion	개독 (*gaedok*)	This is a derogatory expression for Christians, where “*gae*” means “dog” and “*dok*” means “Christians.”	43,341 (10.8%)	13.3%
	개슬람 (*gaeseullam*)	This word is a derogatory expression for Muslims, where “*gae*” means a dog and “*seullam*” means Islam.	8,089 (2.0%)	
	좆슬람 (*jotseullam*)	This word is a derogatory expression for Muslims, where “*jot*” means penis and “*seullam*” means Islam.	2,185 (0.5%)	
Disability	정신병자 (*jeongsinbyeongja*)	This is a derogatory word for people with mental illness.	20,928 (5.2%)	6.3%
	장애인새끼 (*jangaeinsaekki*)	This is a compound of “*jangaein*,” meaning people with disabilities, and “*saekki*,” a derogatory term for a child.	4,430 (1.1%)	

*Note*: The percentage for each keyword denotes the total number of times a given keyword appeared divided by the total number of times any keyword appeared. The total number of times that hateful keywords appeared exceeded the number of posts and comments that we collected (396,496) because individual posts contained more than one keyword. A total of 5,787 (1.5%) posts included more than one hate-related keyword in the same category, and 10,668 (2.8%) posts included hateful keywords in multiple categories.

Table 1 shows that the most prevalent hate-based keyword we encountered was *tteulttak* (틀딱), an insulting term for an old person. Posts with this keyword accounted for 14.0% of all the hateful posts or comments considered in the analysis. The elderly appears to be a major target of online hateful speech by young users of South Korean online communities. Other hate categories with high prevalence included racial hatred (*jjanggae* [짱깨], Korean-Chinese migrants,12.0%), religious contempt (*gaedok* [개독], Protestant believers, 10.8%), and political hatred (*jwappal* [좌빨], progressives, 10.5%).

**Fig 2:**
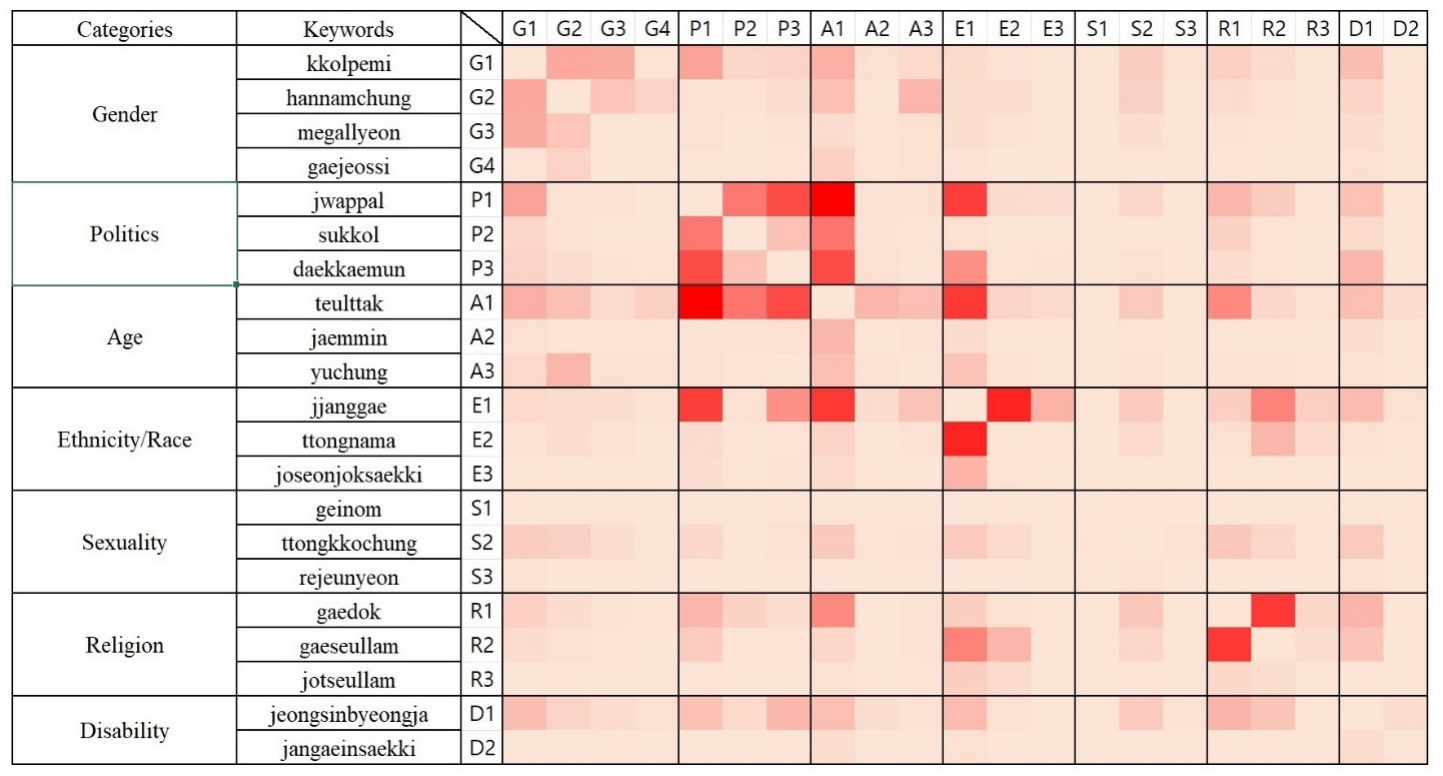
Co-occurrence among hateful keywords.

We further examine the interconnectedness of hateful keywords by revealing the co-occurrence of keywords. Fig 2 illustrates the contextual association among keywords are shown in a two-dimensional heat map. Squares are colored as red when there is a frequent co-occurrence between the two keywords, and pink otherwise.

Fig 2 reveals that hateful keywords included in the same subject category are more likely to co-occur compared to keywords in different categories. Also, a clear combination appears between the subject categories of politics and age as well as ethnicity and politics. More specifically, *teulttak* appears together with *jwappal* and *daekkaemun*, showing that liberals are degraded as being old among young generation within online communities. The degrading terms for Chinese, *jjanggae*, also occur in combination with *jwappal* and *teulttak*, suggesting that liberals have been described as old people who are pro-Chinese.

**Fig 3:**
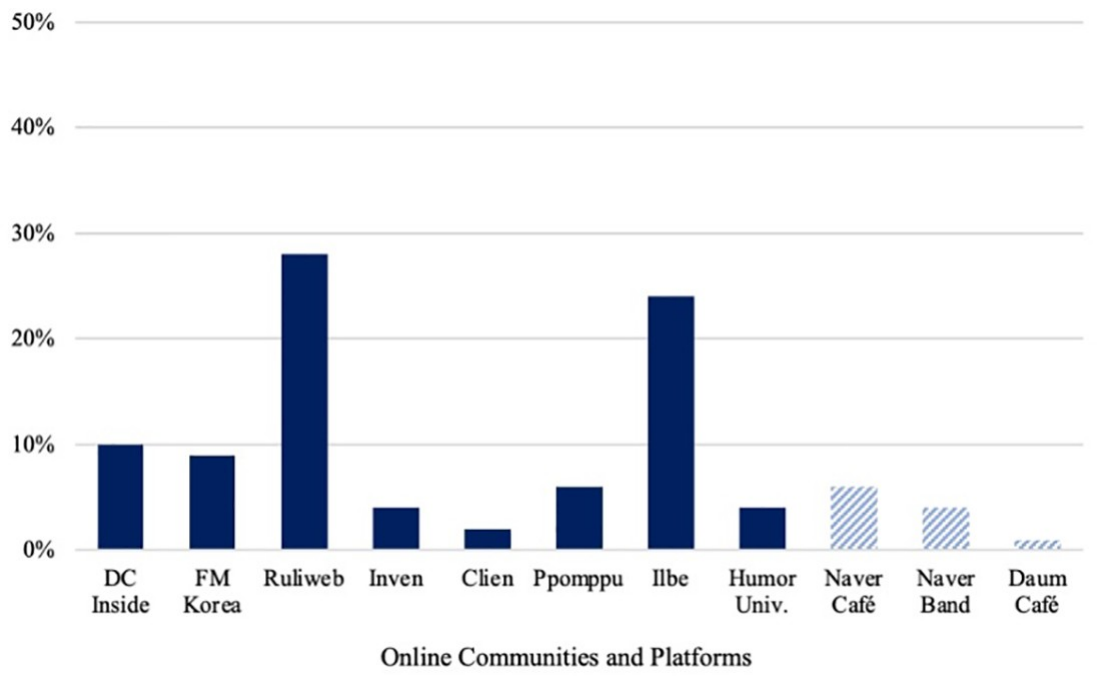
Hateful expressions by online community. *Note*: Bars are dark blue for specialist online communities and light blue for generalist platforms.

Fig 3 shows the prevalence of hateful expressions in the 11 online communities we searched; the figure indicates that hateful posts and comments were not randomly distributed but varied substantially across the different communities. It is worth noting that users of two platforms posted a disproportionately large amount of hate speech: Ruliweb (28.0%), the largest Internet gaming community, and Ilbe Storage (25%), a far-right online community also known as a Korean version of 4chan. The primary users of these two communities are men in their 20s. DC Inside (10.0%), the largest conservative online community in South Korea with more than 20,000 subcommunities (so-called galleries), and FM Korea (9.0%), a spin-off community from DC Inside, also showed higher proportions of hateful expressions than other communities. Our ranking differs from that of the Korea Communications Standards Commission (KCSC), which ranked Ilbe Storage and DC Inside as the top two communities followed by Ruliweb. Readers should be cautious that our ranking is based on the proportion of hateful keywords, whereas the KCSC ranking is based on the number of times each community received a correction request. Although anonymity and the lack of user information make it difficult to make firm statements about the posters of this hate speech, the users of these four platforms are known to be predominately male and conservative. It is also noteworthy that all four online communities that mark high proportions of hateful expressions fall into the category of specialist and less conventional organizations.

**Fig 4:**
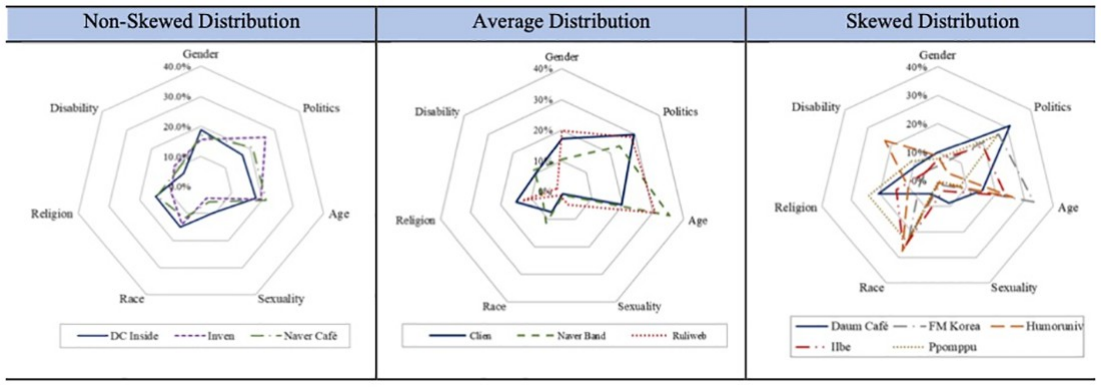
The three typologies of hateful expression by community.

Next, we investigated the frequency of hate terms in the different categories across the different online platforms in Fig 4. We compared radar charts we generated for each of the 11 online communities with the chart of the overall distribution of the seven hate categories shown in Fig 1. Fig 4 provides comparison among different online communities to detect certain patterns, three in particular: (a) a balanced, non-skewed distribution where no hateful category emerges as a dominant one, (b) an average, typical distribution where hateful expressions are distributed primarily in the polarization categories of age and politics, and (c) a skewed distribution where hateful expressions are mostly based on marginalization categories such as race/ethnicity, disability, or religion.

A non-skewed distribution refers to the platforms that hosted hateful posts distributed relatively evenly across all seven hate categories, encompassing both marginalization and polarization. DC Inside was a prime example, with hate speech from all seven domains comprising less than 20% of the total posts. Similarly, Naver Café, the largest community platform in Korea, and Inven, an influential online gaming community, showed a balanced distribution, with the variances between the seven categories being much smaller than other distributions.

The typical distributions were similar to the average distribution reflected in Fig 1. In particular, the combined proportions of hate speech related to age and politics fell between 65% and 80% for the communities characterized by a typical distribution, reflecting polarization in these platforms. Ruliweb, the largest online gaming community in Korea, exemplifies this pattern: Most hateful posts were based on age (30.8%) or political orientation (28.4%). Clien, a major information technology community, and Naver Band, a socially exclusive community platform optimized for mobile users, also show this typical distribution: hatred related to age—19.6% and 35.4%, respectively, and hatred related to politics—29.7% and 23.8%. Whereas average distributions are skewed towards the polarizing categories of age and politics, the skewed distributions reflect the marginalization of minority groups: racial/ethnic minorities, disabled persons, and religious minorities. Posts in five online communities in particular—Daum Café, FM Korea, Humor University, Ilbe Storage, and Ppomppu—featured hateful speech toward vulnerable groups in society.

Consider Daum Café, one of the two community platforms with the most subcommunities (called cafés). Daum Café has more than 1,000,000 members, and on the site, hatred based on religion accounts for about 20.6% of all hateful expressions; on FM Korea, meanwhile, the primary users are men in their 20s to early 30s, and race-based posts account for more than 20% of all hateful posts (22.6%). Humor University, a popular Internet community, also shows a high proportion of hateful posts in the categories of race (27.5%) and disability (22.7%), and on Ilbe Storage, again, over 25% of hateful posts were related to race (26.5%). Finally, Ppomppu, an online community that started as a shopping information website but later turned into a forum for political discussion, also showed significant proportions of hate-based posts skewed towards religion (23.9%) and race (23.7%).

It is noteworthy that although posts among the 11 different communities in this study frequently reflected prejudice against minorities that exceeded the average distribution, community members’ political stances differed across the communities; for example, the primary users of Ilbe are known to be politically conservative, whereas the users of Ppomppu tend toward progressives. As noted earlier, it is highly challenging to capture the average characteristics of the primary users in each platform given the multiplicity of subtopic boards and subcommunities within communities. Users in these communities are typically reluctant to reveal their identities, but it is important to account for both the conformity between the platforms considered and the differences in the distributions of hate speech keywords across the 11 online platforms of this study. We demonstrated that larger communities and platforms, such as DC Inside, Ruliweb, Naver Café, and Naver Band, tend to show balanced or typical distributions but also show that smaller or exclusive communities and platforms are more likely to show skewed distributions that typically target minority populations.

**Fig 5:**
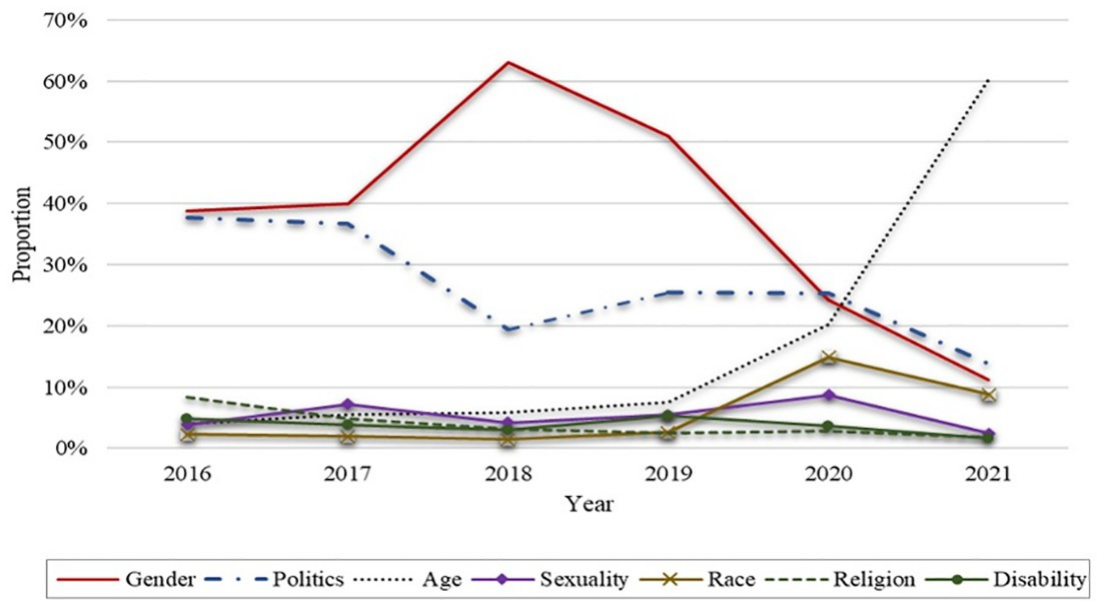
Changing trends in hateful expressions by category: 2016 to 2021.

In particular, in addition to examining the variety and extent of online hateful expressions, we further explored the temporal changes in hate speech during the observational period: 2016–2021. We delved into the three platforms, Ruliweb, FM Korea, and Ilbe Storage, that provide reliable multiyear data under the same layout structure. Fig 5 shows the changing trends in the prevalence of hate domains from 2016 to 2021.

Fig 5 reflects that the topics of hate speech have fluctuated over time. In particular, gender, politics, and age showed the highest prevalence in the 11 platforms we searched, and we identified three patterns. First, the proportion of hateful gender posts increased dramatically in 2018 but decreased substantially after 2019. The #MeToo movement spread rapidly throughout South Korea in 2018 after Prosecutor Seo Ji-hyun reported a case of sexual harassment inside the prosecution’s internal network. Fig 5 indicates that the topics of online hate are heavily influenced by social movements. Second, the proportion of hateful political posts held steady over time, which reflected the polarized nature of politics in South Korea. Finally, age-based hatred spiked in 2021, which corresponded to rising tensions between younger and older generations in South Korea.

**Fig 6:**
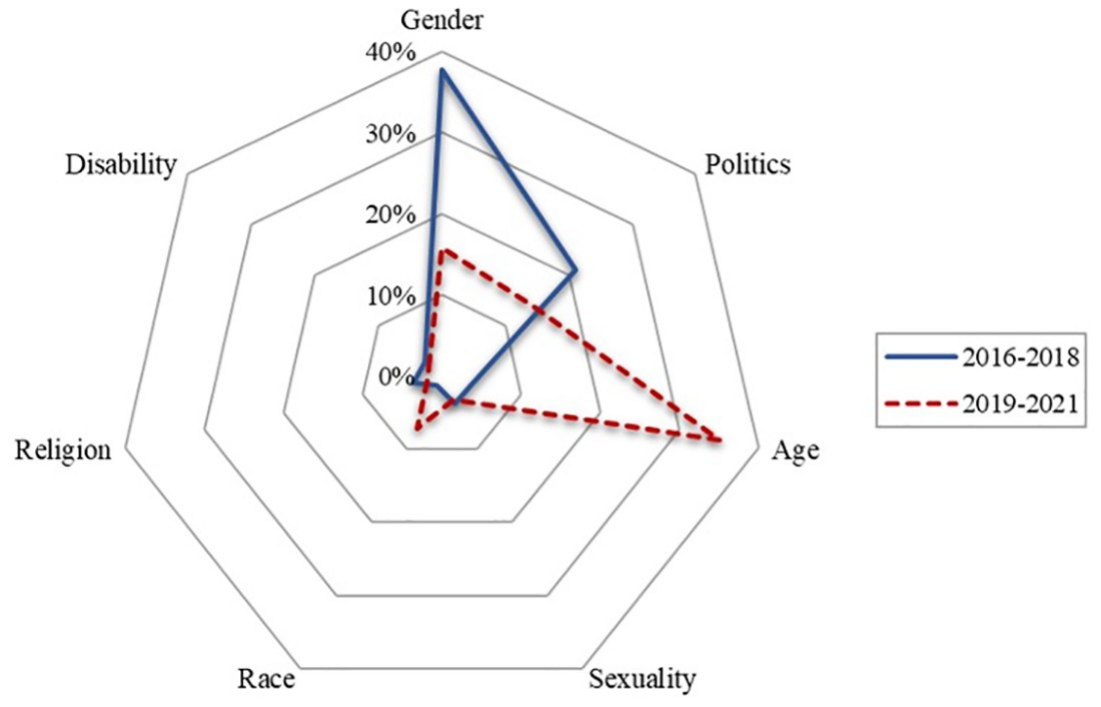
Hateful expressions by category between 2016–2018 and 2019–2021.

Fig 5 also indicates that there was less hate directed toward members of racial or ethnic, sexual, or religious minorities or disabled persons, although race did show fluctuation over time. In particular, race-related hate speech online increased dramatically in 2020 when the COVID-19 pandemic sparked a strong anti-China sentiment. Fig 5 reflects that online hate speech varies with social dynamics. Fig 6 uses a radar chart to visualize how online hatred trends shifted between two different time periods: 2016–2018 and 2019–2021. The figure graphically illustrates how hateful expressions vary over time.

Fig 6 demonstrates a dramatic difference in the proportions of hateful posts between the two distinct periods, 2016–2018 and 2019–2021. From 2016 to 2018, a significant proportion of online hate was based on gender (37.7%), but after 2018, gender-based hateful posts decreased dramatically (15.7%), and age became a dominant topic of hate speech: The proportion of age-based hate speech changed from 4.0% between 2016 and 2018 to 35.1% between 2019 and 2021. Finally, political hate speech also decreased from 21.1% in 2016–2018 to 14.1% in 2019–2021 at the same time that race-based hate posts increased from 1.3% in the former period to 7.1% in the latter. Given that both gender and age categories are related to the dynamics of polarization, our results suggest that the changing trends from 2016–2018 to 2019–2021 is not a dramatic shift from polarization to marginalization but rather a shift within the dynamics of polarization.

## Conclusion

For this study, we analyzed a new data corpus on hatred that we compiled from 11 influential online platforms in South Korea, including both generalist and specialist communities. Based on an initial canvass of keywords related to seven common categories of online hate speech—race, sexuality, religion, age, disability, gender, and politics—we identified notable shifts in patterns over time. For instance, from 2016–2018 to 2019–2021, gender-based hate posts decreased significantly, and age-related hate speech increased dramatically. Our findings indicate wide variation in the topics of hate speech that emerge with shifting sociopolitical trends, for instance the MeToo Movement, which brought a focus to gender. The broad variations we observed over time according to categories of hatred highlight a need for an informed and nuanced understanding of how hatred develops and changes. With our present study, we sought to offer a conceptual framework that is effective in understanding how hatred shifts and evolves in online communities–with a policy implication to find how to combat the effects of this hate speech.

Here, we reflect on the subject of this paper, and we conclude that yes, hatred today is being used to pit all against all. As our research suggests, online hatred engulfs users irrespective of their individual identities and that combating its deleterious effects in a society requires attempting to understand it. We noted in particular that the distinction of polarization and marginalization provides an effective conceptual tool to understand online hatred. It is notable today that polarization is increasing in online forums alongside marginalization that there is little moderation. In this context, we believe there is value in understanding patterns of online hate and being able to report to platform moderators about prejudices to be alert for. With our comprehensive exploration of hateful expression in online communities from an interdisciplinary angle, future studies can build upon our research by developing and testing theories to examine causal links within these communities across the literature of sociology, political science, communication studies, and others.

We note a number of limitations to our study. First, our keywords might not have been representative of the full spectrum of hate speech online. We searched for posts that included hate or prejudice focused on at least one of seven categories of societal hate and prejudice, but it is possible that we overlooked or did not account for different categories of hate and abuse. Furthermore, the keyword-based approach is not free from limitations. Our data may involve inaccuracy given that sentences may not involve any hateful expression even when hateful keywords are included (e.g., in cases when someone criticize people’s usage of hateful keywords in one’s sentences). Using an elaborated model, future studies may detect more nuanced expressions such as (1) non-hateful expressions that have hateful keywords and (2) hateful expressions that do not include any hateful keywords. In addition, future researchers could investigate whether a broader range of categories better represents the spectrum of online hatred today. It would also be useful to better catalog online hate speech directed at combinations of categories, such as women (gender) who are Muslim (religious). Toward the end of broadening the scope, more keywords could be investigated.

Future researchers could also develop techniques for automatically detecting hate speech in online posts with the aim of eliminating abusive speech. Also, considering the aim of our study to show the prevalence of online hate, a computation of the ratio of hatred expression *vis-à-vis* the total number of posts might have been useful. While our keyword-based methods do not allow us to count the total number of all the posts in eleven different layouts for multiple years, future studies can employ a more comprehensive research design to identify hatred expressions within all posts that are uploaded in a particular time-period. We still hope that our findings on the overall scope and dynamics of the current forms of online hateful speech opens up a new avenue of research on hateful expressions in online communities.

We do note that future researchers could use our comprehensive data set to analyze patterns of online hate using multiple other research techniques. For example, topic modeling would allow for better identifying and categorizing specific subjects of hateful expressions. Additionally, network analysis could be applied to examine the relationships among hateful keywords.
